# New Appliances and Things Medical

**Published:** 1899-05-27

**Authors:** 


### NEW APPLIANCES AND THINGS MEDICAL.

[We shall be glad to receive, at our Office, 28 & 29, Southampton Street, Strand, London, W.C., from the manufacturers, specimens of all new preparations and appliances which may be brought out from time to time.]

### SNOWDRIFT DISINFECTANT.

(SNOWDON, SONS, AND CO., LTD., MILLWALL, E.)

SNOWDRIFT Disinfectant is introduced as a concentrated liquid disinfectant and deodoriser, containing the best known disinfectants in which the unpleasant odour is masked by ingredients of a more agreeable character. It is certainly a very pleasant preparation. It is in the form of a thin alkaline emulsion, containing aromatic oils which quite disguise the presence of obnoxious smelling disinfectants. Mixed with water in the proportion of one part in one hundred parts of water the solution makes a very agreeable deodoriser for the hands after operations of a malodorous nature. It will be also found efficacious for sweetening hospital floors, drains, and stables, as well as for many purposes in which an agreeable deodoriser is required.

